# Electroacupuncture prevents astrocyte atrophy to alleviate depression

**DOI:** 10.1038/s41419-023-05839-4

**Published:** 2023-05-29

**Authors:** Si-Si Lin, Bin Zhou, Bin-Jie Chen, Ruo-Tian Jiang, Baoman Li, Peter Illes, Alexey Semyanov, Yong Tang, Alexei Verkhratsky

**Affiliations:** 1 International Joint Research Centre on Purinergic Signalling of Sichuan Province /Research Centre on TCM-Rehabilitation and Neural Circuit, School of Acupuncture and Tuina/Health and Rehabilitation, Chengdu University of Traditional Chinese Medicine, Chengdu, China; 2 Laboratory of Anaesthesia and Critical Care Medicine, Department of Anaesthesiology, Translational Neuroscience Centre, West China Hospital, Sichuan University, Chengdu, China; 3 Department of Forensic Analytical Toxicology, School of Forensic Medicine, China Medical University, Shenyang, China; 4 Rudolf Boehm Institute for Pharmacology and Toxicology, University of Leipzig, Leipzig, Germany; 5 College of Medicine, Jiaxing University, Jiaxing, China; 6 Shemyakin-Ovchinnikov Institute of Bioorganic Chemistry, Russian Academy of Sciences, Moscow, Russia; 7 Acupuncture and Chronobiology Key Laboratory of Sichuan Province, Chengdu, China; 8 Faculty of Biology, Medicine and Health, The University of Manchester, Manchester, UK; 9 Achucarro Centre for Neuroscience, IKERBASQUE, Basque Foundation for Science, Bilbao, Spain

**Keywords:** Astrocyte, Depression

## Abstract

Astrocyte atrophy is the main histopathological hallmark of major depressive disorder (MDD) in humans and in animal models of depression. Here we show that electroacupuncture prevents astrocyte atrophy in the prefrontal cortex and alleviates depressive-like behaviour in mice subjected to chronic unpredictable mild stress (CUMS). Treatment of mice with CUMS induced depressive-like phenotypes as confirmed by sucrose preference test, tail suspension test, and forced swimming test. These behavioural changes were paralleled with morphological atrophy of astrocytes in the prefrontal cortex, revealed by analysis of 3D reconstructions of confocal Z-stack images of mCherry expressing astrocytes. This morphological atrophy was accompanied by a decrease in the expression of cytoskeletal linker Ezrin, associated with formation of astrocytic leaflets, which form astroglial synaptic cradle. Electroacupuncture at the acupoint ST36, as well as treatment with anti-depressant fluoxetine, prevented depressive-like behaviours, astrocytic atrophy, and down-regulation of astrocytic ezrin. In conclusion, our data further strengthen the notion of a primary role of astrocytic atrophy in depression and reveal astrocytes as cellular target for electroacupuncture in treatment of depressive disorders.

## Introduction

Astrocytes are principal homeostatic cells of the central nervous system (CNS) supporting nervous tissue at all levels of organisation, from molecular to organ-wide [1]. Astrocytes present a complex spongiform morphology formed by numerous tiny peripheral processes known as leaflets emanating from primary and higher order processes known as branches [2, 3]. Astrocytic leaflets establish contacts with synapses and form synaptic cradle to foster and maintain synaptic connectivity [4, 5]. In particular, leaflets are densely populated by diverse plasmalemmal Na^+^-dependent transporters responsible for neurotransmitter and ion homeostasis in the synaptic cleft [6, 7]. In addition, astrocytes secrete numerous factors modulating synaptic function [8]. The leaflets demonstrate high degree of physiological morphological plasticity, which also modulates synaptic transmission [9]. In ageing and various forms of neuropathology shrinkage of leaflets leads to reduced homeostatic support, aberrant neurotransmitters spillover, and impaired synaptic plasticity [9–11]. Astrocytic atrophy with reduced leaflets presence is prominent in brain ageing [10] and is implicated in pathophysiology of a wide range of neurological diseases [12], including neurodegenerative disorders [13, 14], epilepsy [15], addiction [16], and mood disorders [17]. Astrocytic atrophy is the leading histopathological signature of mood disorders in response to stress, in particular in the major depressive disorder (MDD) [18–20] and post-traumatic stress disorder [21].

Pharmacological management of MDD remains a challenge, with many patients being resistant to traditional antidepressants. Electroacupuncture (EA) stimulation is employed as an effective and safe therapy to treat multiple diseases including inflammation [22, 23], Parkinson’s disease [24], insomnia [25–27], anxiety [28] and depression [29–31]. The EA at selective acupoints Dazhui (DU14) and Baihui (DU20) was shown to restore inhibitory/excitatory balance and improve cognition in mouse models of Alzheimer’s disease [32]. Several studies indicated that EA affects astrocytes by modulating expression of glial fibrillary acid protein (GFAP) [33] and production of fibroblast growth factor 2 (FGF2), the latter being beneficial for alleviating depressive-like behaviour [31].

In the present study, using high-resolution morphological reconstructions we analysed protoplasmic astrocytes in the prefrontal cortex of healthy control mice and mice, which developed depressive-like behaviours following exposure to chronic unpredictable mild stress (CUMS). We found that CUMS lead to a significant morphological atrophy of astrocytes associated with down-regulation of plasmalemmal-cytoskeletal linker Ezrin. Treatment of these mice with EA or with classical anti-depressant fluoxetin normalised astrocytic morphology, increased expression of Ezrin, and alleviated depressive-like phenotype. Our work suggests that EA may provide a potential non-pharmacological therapeutic tool targeting astrocyte atrophy in MDD.

## Results

### Validation of the depression model: CUMS triggers anhedonia and depressive-like behaviours

**Fig. 1:**
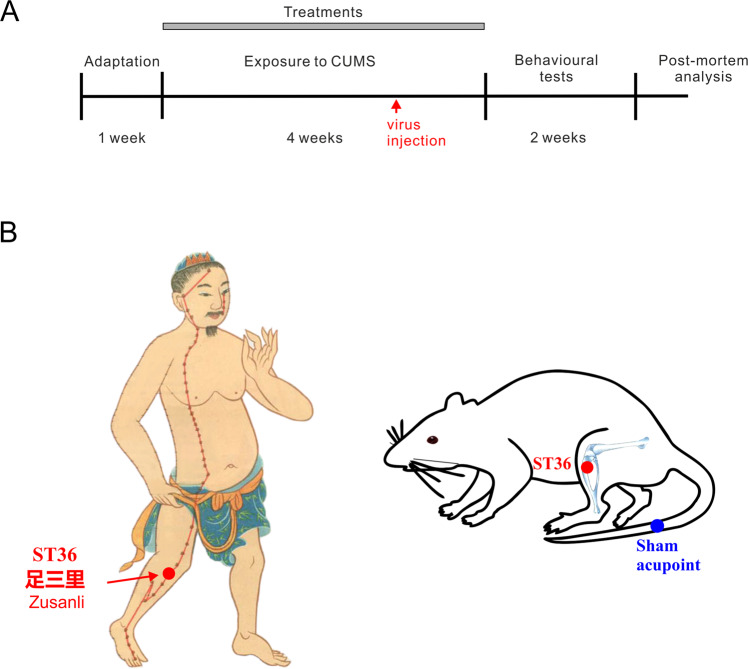
Experimental design. **A** Flow diagram of the experimental procedure. **B** Location of acupoints used in the study. The man acupoint image is modified based on an old image created during Ming dynasty.

**Fig. 2:**
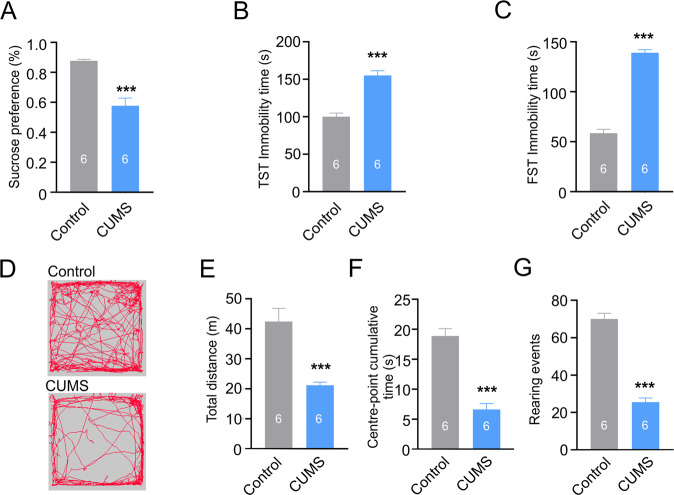
The CUMS regimen triggers depressive-like phenotypes in mice. **A**–**C** CUMS reduced sucrose intake and prolonged the TST and FST immobility time. **D** CUMS affected exploratory behaviour in an open field test; Representative running trace in open field test, the observation time was 10 min. **E**, **F**, **G** Total running distance, centre-point cumulative duration, and number of rearing events. All data are presented as mean ± sem. *p < 0.05, **p < 0.01, ***p < 0.001. The number of experiments is indicated on each column.

Exposure of rodents to CUMS and its variants is widely used to model depression [20, 34–36]. The experimental design including the timeline of the experimental protocol and positions of acupoints used in this study are shown in Fig. 1. Treatment of mice with CUMS regimen for 4 weeks resulted in a significant decrease in sucrose preference reflecting the development of anhedonia, one of the key signs of depression in patients [37–39]. The sucrose consumption decreased from 0.88 ± 0.01 in control to 0.58 ± 0.05, *p* < 0.001, *n* = 6 in mice exposed to CUMS protocol (Fig. 2A). Similarly, exposure to CUMS regimen affected mouse behaviours as revealed in a series of tests. The immobility time in both tail suspension test (TST) and force swimming test (FST) was significantly prolonged (from 100.1 ± 4.8 in control to 155.1 ± 6.3, *p* < 0.001, *n* = 6 in CUMS group and from 58.4 ± 4.0 in control to 139.1 ± 3.09, *p* < 0.001, *n* = 6 in CUMS group respectively; Fig. 2B, C). In the open field test CUMS decreased exploratory behaviour as demonstrated by a decrease in total running distance, centre-point cumulative duration and rearing frequency (from 42.488 ± 4.358 m in control to 21.153 ± 1.09 m, *p* < 0.001, *n* = 6; from 18.9 ± 1.2 s in control to 6.64 ± 1 s, *p* < 0.001, *n* = 6; from 70 ± 3.1 in control to 25.5 ± 2.3, *p* < 0.001, *n* = 6 respectively; Fig. 2D–G). Together, these data indicate that CUMS induces depressive-like behaviours.

### Depression is associated with astrocytic atrophy: CUMS reduces morphological profiles and decreases expression of ezrin

**Fig. 3:**
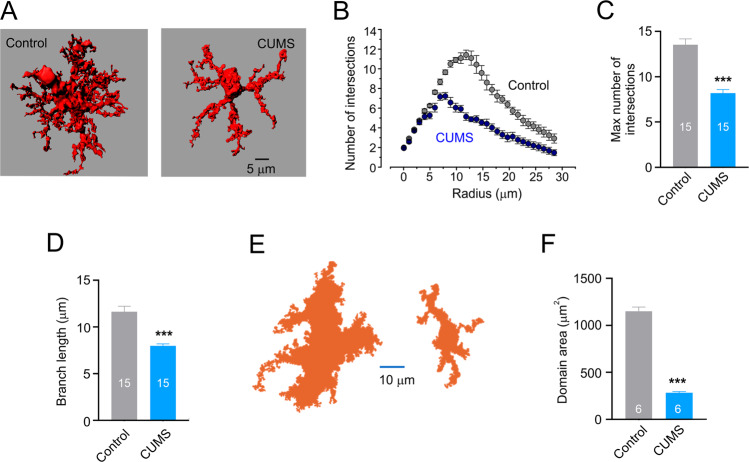
CUMS induces morphological atrophy in PFC astrocytes. **A** Representative 3D reconstruction of astrocyte in control and CUMS groups. **B** Sholl analysis of astrocytic morphology for control and CUMS groups shows the number of intersections of astrocytic branches with concentric spheres centred in the middle of cell soma. **C** Maximal number of intersections for astrocytes in control and CUMS groups. **D** Average length of astrocytic processes in control and CUMS groups. **B**–**D** *n* = 15 for each group. **E** Representative examples of astrocytic territorial domains obtained as a projection of astrocytes along the z-axis projection for control and CUMS animals. **F** Average astrocytic domain for control and CUMS group. All data are presented as mean ± sem. *p < 0.05, **p < 0.01, ***p < 0.001. The number of experiments is indicated in each column.

To access morphological changes in astrocytes following CUMS we labelled astrocytes in the prefrontal cortex (PFC) of mice by stereotactic injection of AAV5·gfaABC1D·mCherry [40], which allowed reliable morphometric analysis (Fig. 3). The three-dimensional (3D) reconstructions of mCherry-labelled healthy and stressed astrocytes were made from z-stacks of images obtained with confocal microscopy (Fig. 3A). Astrocytic 3D-reconstructions were subjected to Scholl analysis inbuilt in the Imaris software. Exposure to CUMS regimen resulted in a substantial decrease in astrocytic size and complexity (Fig. 3). The maximal number of intersections was significantly decreased from 13.5 ± 0.9 in healthy to 8.2 ± 0.4 in depressed animals (*p* < 0.001; *n* = 15, Fig. 3B, C), while and the length of astrocytic branches decreased from 11.6 ± 0.7 µm in control to 8.0 ± 0.2 µm, *p* < 0.001 in CUMS mice; *n* = 15 (Fig. 3D). Similarly, exposure to CUMS protocol significantly reduced the size of astrocytic territorial domain (from 1151.6 ± 44.3 µm^2^ in control to 283.7 ± 13.3 µm^2^ in CUMS animals, *p* < 0.001; *n* = 6, Fig. 3E, F).

**Fig. 4:**
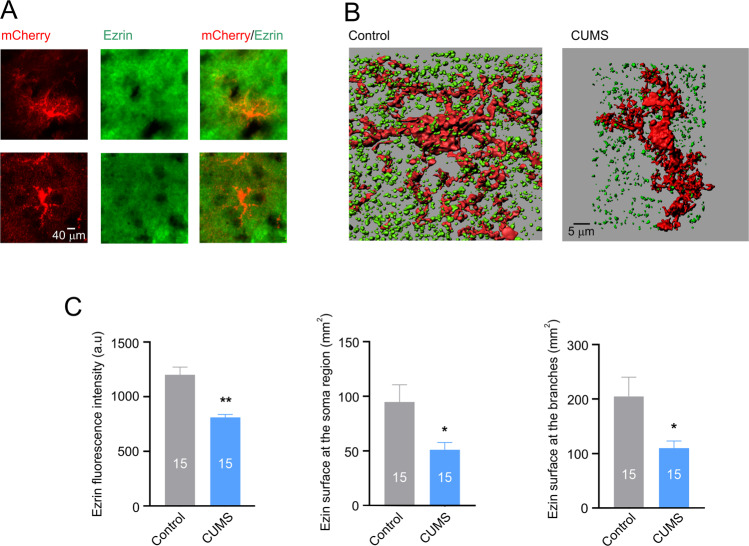
Chronic stress decreases ezrin association with astrocytes. **A** Images of astrocytes labelled with AAV-GfaABC1D-mCherry and immunostained for ezrin. **B** Representative 3D-reconstruction of astrocytic profiles (red) with Ezrin puncta (green) for control and CUMS groups. **C** Average fluorescence intensity of ezrin and surface area of ezrin puncta associated with soma and branches of 3-D reconstructed astrocytes. All data are presented as mean ± sem. *p < 0.05, **p < 0.01, ***p < 0.001 compared to control group. The number of experiments is indicated in each column.

Ezrin, a member of ezrin-radixin-moesin (ERM) family tethers actin filaments to the plasma membrane, and is essential for the formation and structural plasticity of astrocytic leaflets and astrocyte-synaptic interactions [41, 42]. We quantified ezrin expression with immunocytochemistry, applied to the PFC preparations from mice subjected to mCherry astrocytic labelling (Fig. 4A). The fluorescence of ezrin associated with mCherry-positive astrocytic profiles was significantly decreased in depressed animals (from 1201.2 ± 73.4 in control to 813.0 ± 28.4 in CUMS-treated animals, *p* < 0.01; *n* = 15, Fig. 4B, C). The number and surface area of ezrin-positive puncta uniformly decreased around the soma and branches of depressed animals (from 94.7 ± 16.3 µm^2^ to 51.0 ± 6.8 µm^2^, *p* = 0.016, *n* = 15, and from 204.8 ± 36.6 µm^2^ to 110.2 ± 13.5 µm^2^, *p* = 0.018, *n* = 15 respectively, Fig. 4D, E).

### Fluoxetine and EA prevent depressive-like behaviours induced by CUMS

**Fig. 5:**
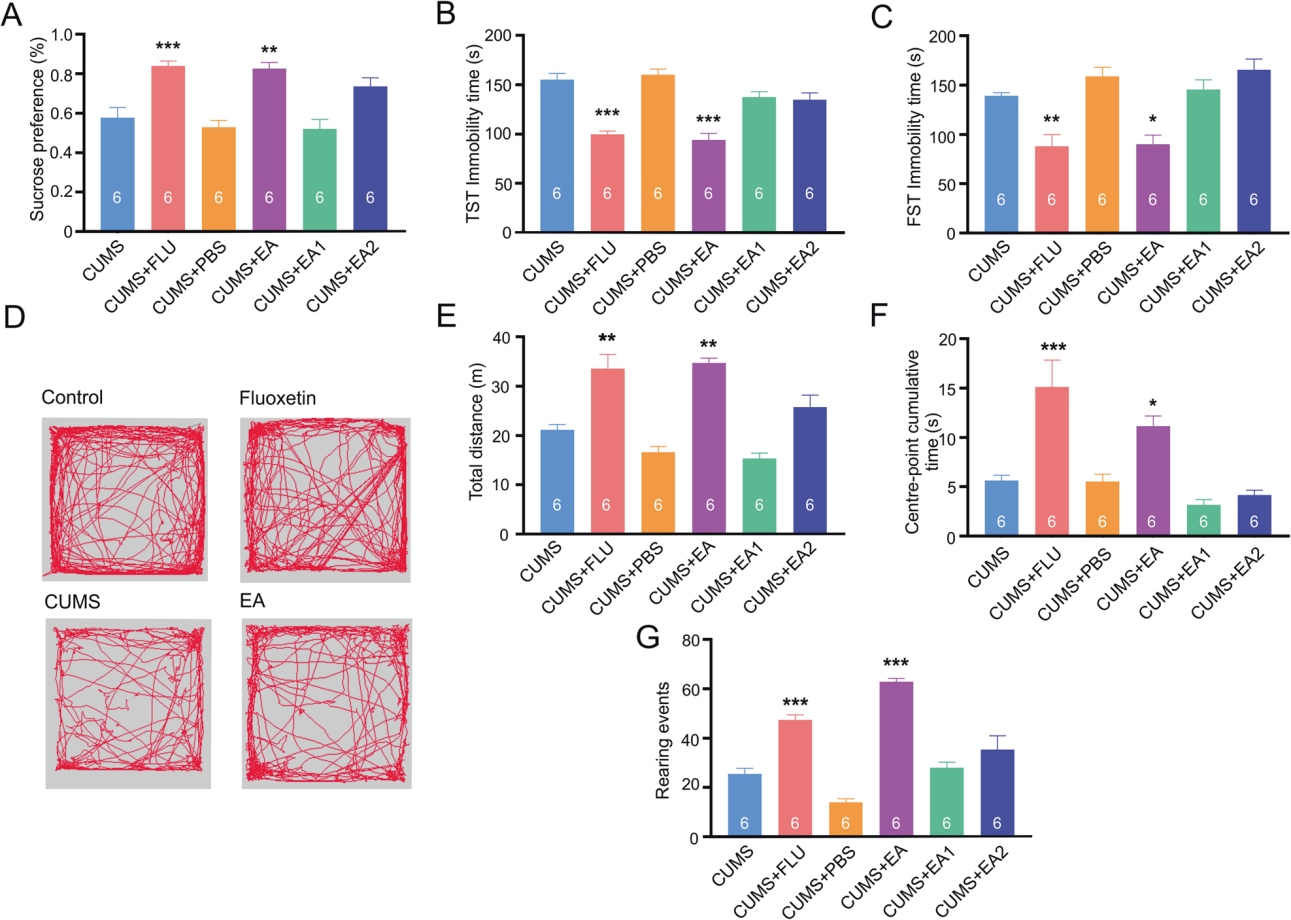
Treatments with fluoxetine and EA prevents chronic stress-induced development of depressive-like behaviours. **A** Sucrose preference test. **B** TST immobility time. **C** FST immobility time. **D** Representative running trace in open field test, the observation time was 10 min. **E**–**G** Average values for total distance, centre-point time, and the number of rearing events. Experimental groups are indicated on the graphs. All data are presented as mean ± sem. *p < 0.05, **p < 0.01, ***p < 0.001 compared to control group. The number of experiments is indicated in each column.

In this study, we compared the anti-depressant efficacy of EA in the specific acupoint with the action of classic anti-depressant drug fluoxetine (a.k.a Prozac). We found that treatment with fluoxetine as well as treatment with EA at Zusanli acupoint (足三里, ST36) used in traditional Chinese medicine (TCM) for therapy of depression fully prevented the development of depressive-like behaviours in CUMS exposed mice (Fig. 5). In particular, in animals treated with fluoxetine and EA sucrose consumption was significantly higher compared to the CUMS group (Fig. 5A; fluoxetine: 0.8 ± 0.03 vs. 0.6 ± 0.05 in CUMS, *p* < 0.001 EA: 0.8 ± 0.03 vs. 0.6 ± 0.05 in CUMS; *p* < 0.01, *n* = 6). Similarly, fluoxetine and EA prevented the development of depression-like behaviours in TST and FST (Fig. 5B, C); both treatments also prevented a decrease in exploratory behaviours in the open field test (Fig. 5D–G). The efficacy of fluoxetine and EA were similar without any significant defences in tests readouts. At the same time, neither injection of PBS nor sham electroacupuncture (EA1, EA2: acupuncture without electrical stimulation or acupuncture in clinically irrelevant acupoints, see methods section) were effective in preventing the development of depressive-like behaviours in mice exposed to the CUMS protocol.

### Fluoxetine and EA prevent astrocyte atrophy and increase the presence of ezrin

**Fig. 6:**
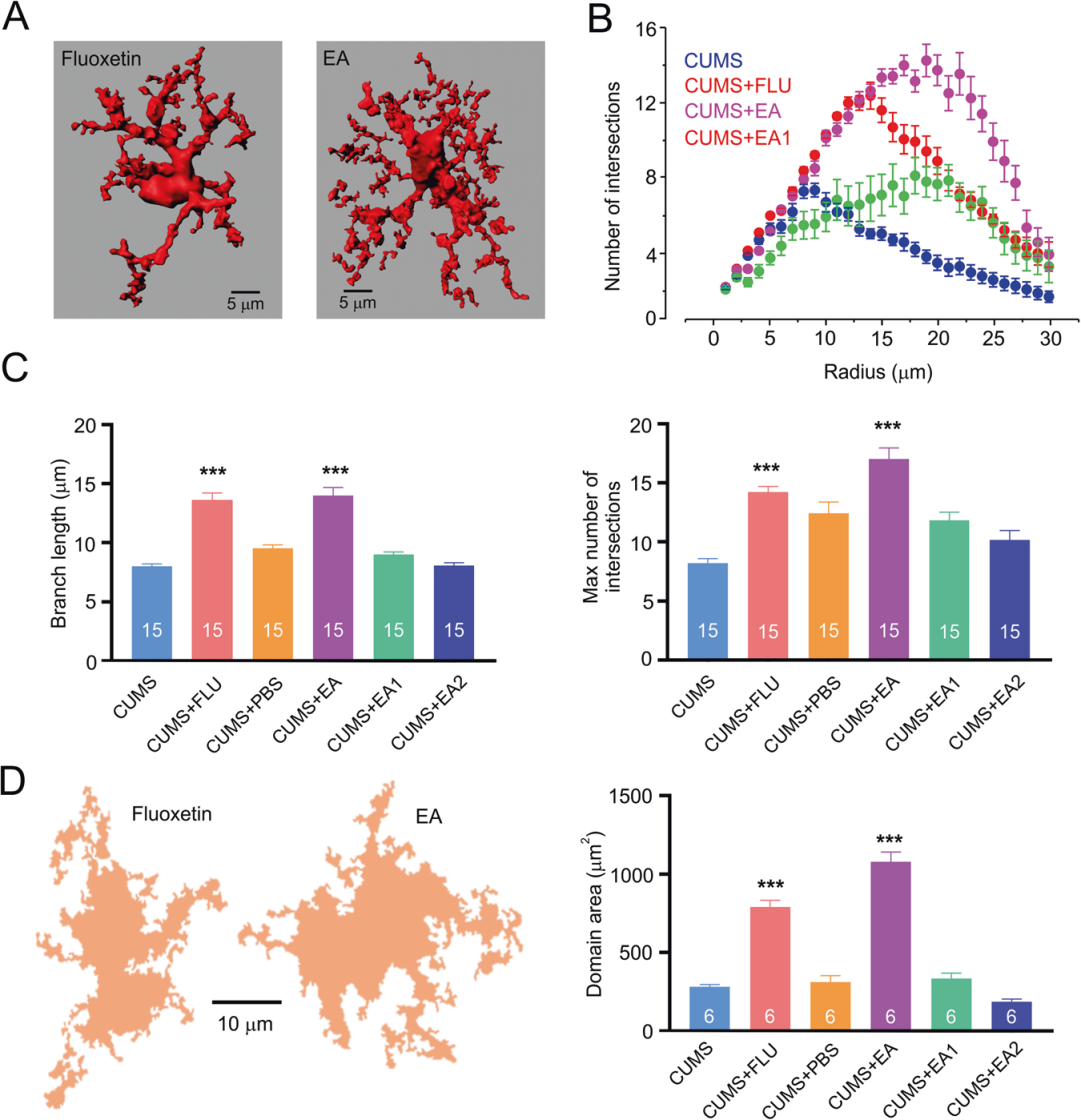
Treatment with fluoxetine and EA prevents chronic stress-induced development of astrocytic atrophy. **A** Representative 3D reconstruction of astrocyte in CUMS + fluoxetine and CUMS + EA groups. **B** Sholl analysis of astrocytic morphology for CUMS, CUMS + fluoxetine, CUMS + EA, and CUMS + EA1 groups. **C** Maximal branch length and number of intersections; experimental groups are indicated on the graph. **B**, **C** *n* = 15 for each group. **D** Representative examples of astrocytic territorial domains obtained as a projection of astrocytes along the z-axis projection for CUMS + fluoxetine and CUMS + EA. The average domain area across various experimental groups is shown on the left. Experimental groups are indicated on the graphs. All data is presented as mean ± sem. *p < 0.05, **p < 0.01, ***p < 0.001 compared to control group. The number of experiments is indicated in each column.

**Fig. 7:**
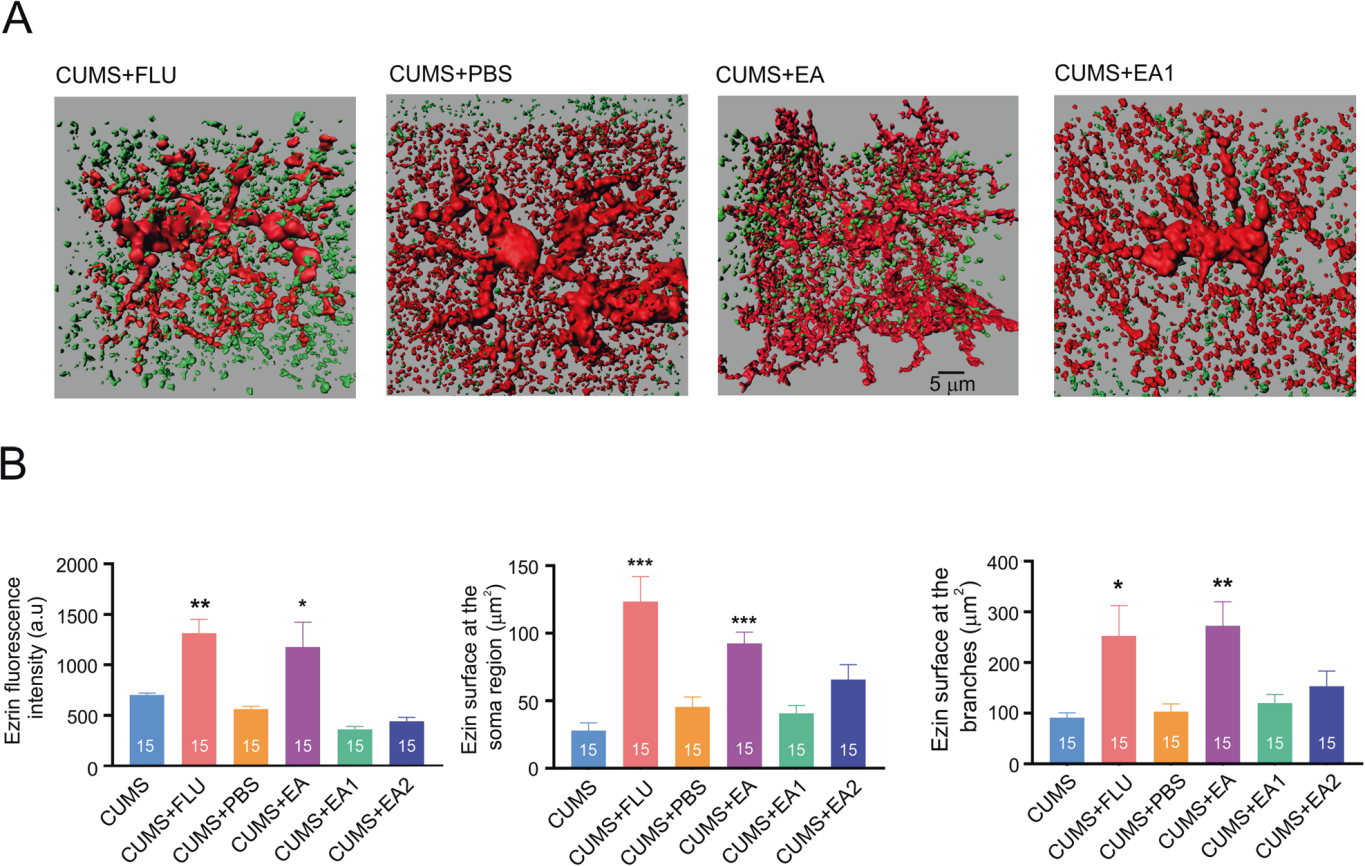
Treatment with fluoxetine and EA preserves ezrin-astrocyte association under CUMS regimen. **A** Representative 3D-reconstruction of astrocytic profiles (red) with Ezrin puncta (green) for CUMS + fluoxetine, CUMS + PBS, CUMS + EA, and CUMS + EA1 (sham acupuncture procedure). **B** Average fluorescence intensity of ezrin and surface area of ezrin puncta associated with soma and branches of 3-D reconstructed astrocytes across various experimental groups. Experimental groups are indicated on the graphs. All data are presented as mean ± sem. *p < 0.05, **p < 0.01, ***p < 0.001 compared to control group. The number of experiments is indicated in each column.

In parallel with preventing the development of depression-like behaviours, treatment with fluoxetine and exposure to EA averted CUMS-induced astrocytic atrophy (Fig. 6) and CUMS-induced decrease of astrocyte-associated ezrin (Fig. 7). In particular, in the animals subjected to fluoxetin or EA treatments maximal number of intersections and length of astrocytic branches was significantly higher than in the CUMS group (Intersections; fluoxetine: 14.2 ± 0.5 vs. 8.2 ± 0.4 in CUMS; *p* < 001, *n* = 15; EA: 17 ± 1.0 vs. 8.2 ± 0.4 in CUMS, *p* < 0.001, *n* = 15; length of branches fluoxetine: 13.6 ± 0.6 vs. 8.0 ± 0.2 in CUMS; *p* < 0.001, *n* = 15; EA: 14.0 ± 0.7 vs 8.0 ± 0.2 to *p* < 0.001; *n* = 15; Fig. 6A–C). Likewise, both fluoxetine and EA prevented CUMS-induced decrease in astrocytic territorial domain (fluoxetine: 790.5 ± 43.2 vs. 283.7 ± 13.4 in CUMS; *p* < 0.001, *n* = 6; EA: 1078.0 ± 62.3 μm^2^ vs. 283.7 ± 13.4 μm^2^ in CUMS; *n* = 6; Fig. 6D). Neither injections of PBS, nor sham EA were able to prevent CUMS-induced changes in astrocytic morphology (Fig. 6B–D).

Similar effects fluoxetin and EA exerted on ezrin association with astrocytes (Fig. 7). In animals receiving fluoxetin or EA treatment the fluorescence intensity of ezrin associated with astrocytic domain was significantly higher than in CUMS only group (fluoxetine: 1248.4 ± 140.5 vs. 703.8 ± 19.4 in CUMS; *p* < 0.01 *n* = 15; EA: 1176.14 ± 254.6 vs. 703.8 ± 19.4 in CUMS, *p* < 0.05; *n* = 15, Fig. 7B). Immunolabelled ezrin puncta increased homogeneously in soma and branches areas (Fig. 7B). Again, neither injections of PBS, nor sham EA affected ezrin levels.

## Discussion

### Chronic stress induces astrocytic atrophy: pathophysiological relevance for depression

Using high-resolution morphometric analysis we confirmed previous observations showing that chronic stress causes morphological atrophy of astrocytes in several areas of the brain including PFC [20, 43, 44], which was the focus of our study. The high-resolution morphometry of astrocytes in situ requires cytosolic labelling with fluorescent probe; the latter could be either injected into the cells of interest through microelectrode (for example Lucifer yellow [40]), or patch-pipette (for example Alexa Fluor 594 [45]) or expressed under the control of astroglia-specific promoter (such as *Aldh1l1* [20] or *Gfap* [43]). In our study, we used mCherry and *Gfap* promoter for a viral transfection (AAV5-gfaABC1D-mCherry; see also [40]). Astrocytic atrophy was quantified by Scholl analysis, which showed significant decrease in the number of intersections, decreased length of astrocytic branches and reduced area of astrocytic territorial domains (Fig. 2). In addition, chronic stress affected astrocytic presence of ezrin, as judged by immunocytochemistry—in animals exposed to CUMS number of labelled ezrin puncta associated with individual astrocytes (both at the soma and at branches) was significantly decreased (Fig. 3). Ezrin is a linker of plasmalemma and cytoskeleton, and it is critical for formation and morphological plasticity of astrocytic leaflets [41, 46]; decrease in ezrin leads to a decrease in the number and/or volume of leaflets. The leaflets are part of idiosyncratic morphology of protoplasmic astrocytes, which gives them spongiform or bushy appearance [3]. These leaflets are characterised by an extremely high surface-to-volume ratio and absence of organelles [3, 47], which stipulates specificity of ionic signalling in these compartments (predominance of Ca^2+^ entry and prominence of Na^+^ signalling [48, 49]). The leaflets are closely associated with synapses and, by virtue of high density of plasmalemmal homeostatic transporters, sustain and regulate synaptic transmission [2].

Astroglial atrophy, asthenia and loss of function is a distinct astrogliopathological entity observed in many neurological diseases [12]. Decreased astrocytic presence in the brain active milieu has multiple consequences. Shrunken astrocytes open diffusional channels and facilitate volume transmission [50]; at the level of single synapses, reduced presence of astrocytic leaflets impairs clearance of glutamate, leading to increased availability (with possible excitotoxic repercussions) and spillover of the latter thus affecting synaptic plasticity [10]. Furthermore, reduced glial presence limits homeostatic support, provision of glutamine, K^+^ buffering, control over pH and supply of scavengers of reactive oxygen species [2, 51]. To the contrary, when the growth of astrocytic leaflets is promoted, the support of the synaptic transmission is increased. Astrocytes are characterised by high degree of morphological plasticity and various environmental factors are known to increase astrocytic domains, mainly through an increase of leaflets presence. These environmental factors include, for example, enriched environment and physical activity [52, 53], dieting [45, 54] and other life style factors, positively impacting cognitive abilities [55]. In the context of mood disorders astrocytic atrophy can contribute to the aberrant neurotransmission and affect neuronal survival and overall functional performance. Indeed, specific manipulation with astrocytes and astrocytic homeostatic cascades can induce depression-like behaviours, whereas treatment with antidepressants rescues these behaviours and reverses astrocytic atrophy [56, 57]. Here we demonstrate that EA acts very similarly to chemical antidepressants and prevents development of depressive behaviours and astrocytic atrophy in the PFC of mice exposed to CUMS.

#### Electroacupuncture acts on astrocytes to prevent depression?

Acupuncture was used in Traditional Chinese Medicine as a therapeutic manipulation for last 3000 to 4000 years [58]; with the first codex formalising acupuncture treatments (*The Yellow Emperor’s Classic of Internal Medicine*) was published around 100 BC [59]. Acupuncture is now used worldwide for treatment of various medical conditions [22, 60–62]. Acupuncture is employed for therapies of neuropsychiatric diseases, including depression [63, 64] and anxiety [65]. In particular, 6 weeks of acupuncture at acupoints Baihui (GV20) and Zusanli (ST36), or Taichong (LR3), Sanyinjiao (SP6), Neiguan (PC6), and Shenmen (HT7), was as effective as oral fluoxetine in treating depression, although acupuncture showed better response and improvement rates [66]. Similarly, acupuncture at ST36 and CV4 acupoints was effective in alleviating depressive-like behaviours in animal models [67, 68]. In our present study, we chose to perform EA at the Zusanli (ST36, 足三里, or point of longevity) acupoint, known to be linked to the brain. Acupuncture at this acupoint affects brain metabolism of glucose [69], modulates excitation-inhibition balance in cortex [70] and triggers responses of several brain areas including the anterior cingulate cortex (ACC), ventrolateral prefrontal cortex (VLPFC), occipital cortices, somatosensory cortex, and midbrain [71, 72].

We found that EA, as well as treatment with fluoxetine fully prevents the development of depressive-like behaviours in mice subjected to chronic stress (Figs. 2, 5). To elucidate the possible brain cellular target of the EA, we performed in depth analysis of fine morphology of cortical astrocytes, known to become atrophic in depression. Exposure of mice to the CUMS regimen led, as expected, to a significant decrease in astrocytic size and complexity (Fig. 3), which were fully prevented by both fluoxetine treatment and EA at acupoint ST36. This effect was specific as sham acupuncture was ineffective. Treatment with EA (as well as with fluoxetine) prevented decrease in ezrin associated with astrocytic structures. We may therefore suggest that chronic stress leads to a retraction of astrocytic leaflets and decrease of astrocyte-synaptic association, which in turn impairs synaptic transmission and plasticity, as has been demonstrated in ageing and various neuropathologies [10, 45, 50, 54] (responsible for generation of depressive-like behaviours. Our data are supported by recent observation demonstrating that EA restores expression of astrocyte-specific glutamate transporter EAAT2 following chronic stress; this restoration developed in parallel with amelioration of depressive-like behaviours [73, 74].

What are the mechanisms translating acupuncture into changes in astrocytic morphology and functional boost which protects against depressive changes instigated by chronic stress? At this stage we can only speculate. As we demonstrated in this study, classic anti-depressant fluoxetine rescues stress-induced depressive behaviours as well as prevents astrocytic atrophy. Fluoxetine acts on astrocytes either through inhibition of serotonin transporter SERT/ SLC6A4 (which, however, is mainly expressed in neurones) or through acting directly on astrocytic 5-HT_2B_ receptors. Activation of these receptors by fluoxetine triggers several signalling cascades [75, 76], and induces transactivation of epidermal growth factor receptors (EGFR), which, in turn, recruits MAPK/ERK or PI3K/AKT downstream cascades to regulate expression of several genes (such as Ca^2+^-dependent phospholipase A2, cPLA2, subtype 2 of adenosine deaminases acting on RNA’s, ADAR2, or subtype 2 of kainate receptors, GluK2) related to mood disorders [77]. Fluoxetine also normalises interstitial pH which is affected in mood disorders by phosphorylating and stimulating astrocytic Na^+^-H^+^ transporter NHE/SLC9a1 [78]. Whether EA may act through the similar cascade requires further investigation. We may only note that EA can also act though stimulation of the subcortical nuclei [79] by activation noradrenergic or dopaminergic systems, known to stimulate specific astrocytic receptors to induce various signalling cascades [80–82].

## Conclusion

In summary, we demonstrated that EA prevents the development of depression-like behaviours in mice exposed to chronic stress. At cellular and molecular levels, EA prevents morphological atrophy of astrocytes (which is a leading histopathological hallmark of depression) as well as the decrease in astrocyte-associated ezrin that controls fine morphology of these cells. Our study therefore provides, for the first time, experimental evidence for an idea that EA boosts astrocytic presence in the brain active milieu thus protecting it against stress-induced pathological deformations resulting in depressive behaviours.

## Materials and methods

### Animals

All experiments were performed on C57BL/6 mice (obtained from Chengdu Dossy Experimental Animal Co., Chengdu, China); the mice were 7 weeks old at the beginning of the experimental protocol, which lasted 7 weeks (Fig. 1A). All mice were adapted to the standard laboratory conditions (24 ± 2 °C room temperature and 65 ± 5% humidity on 12/12 h light-dark cycles) with drinking water and food available ad libitum. No statistical methods were used to pre-determine sample sizes, but our sample sizes are similar to those reported in previous publications [15, 20, 36]. The experimental procedures were made in accordance with the National Institute of Health Guidelines for the Care and Use of Laboratory Animals and approved by the Animal Ethics Committee of Chengdu University of Traditional Chinese Medicine (protocol code, AM3520, 8 May 2019).

### Chronic unpredictable mild stress (CUMS) regimen

Mice were exposed to the random sequence of stressors during each 24-h period for 4 weeks, as previously described [34, 83]. These stressors included water and food deprivation (12 h), cage tilt 45° (12 h), group housing (12 h), swimming in 4 °C water (5 min), foot shock (1 mA, 5 min), noise (120 dB for 3 h), tail suspension (5 min), damp bedding (12 h), cage shaking (40/min for 5 min), and restraint (1 h).

### Experimental groups and treatments

We established 7 experimental animal groups (to which mice were randomly assigned): (i) control group; which did not receive any interventions; (ii) CUMS groups—animals exposed to CUMS only; (iii) CUMS + fluoxetine group—animals exposed to CUMS and weekly injections (for all period of CUMS treatment) of fluoxetine at 10 mg/kg; (iv) CUMS + PBS group, animals exposed to CUMS and daily injections of PBS (0.2 ml); (v) EA group animals exposed to EA daily and (vi-vii) sham EA1 and EA2 groups exposed to sham acupuncture daily.

EA was administered at roughly the same time of the day (10:00 a.m. to 11:00 a.m.) to awake animals, immobilised by two Velcro brand hooks and loop fasteners as well as additional tapes fixed to a wooden block for the duration of EA [84]. EA was delivered to the therapeutically relevant Zusanli acupoint (ST36; located at the knee, about 2 mm for mice below the fibular head). An electrical current of 0.5 mA and a frequency of 2 Hz was delivered for 30 min, by an acupoint nerve electrostimulator (HANS-200, Nanjing Jisheng Medical Technology Co., Jiangsu, China). EA was applied through stainless steel needles (2.5 cm long, 0.25 mm diameter; Hwato-Med. Co., Jiangsu, China), introduced 2–3 mm deep below the skin at ST36 unilaterally. Sham treatments were as follows. In the sham EA1 group the needle was inserted but the electrical stimulation was not applied. In the sham EA2 sham group the needle was positions at the non-acupoint at the tail [85].

### Behavioural tests

#### Sucrose preference test

The sucrose preference test is a reward-based test and a measure of anhedonia, as previously described [86]. The mice were singly caged for 3 days and given two 50 mL bottles containing water or water-based 1% sucrose solution (wt/vol), respectively. The bottle positions were switched daily to avoid a side bias. Following a 24 h period of water and food deprivation, the preference for sucrose or water was determined overnight. Sucrose preference (%) was quantified as (vol sucrose/(vol sucrose + vol water)) × 100%.

#### Tail suspension test

The tail suspension test is a behavioural despair-based test assessing the duration of immobility of mice subjected to inexorable conditions, as previously described [87]. Each mouse was suspended by its tail at a height of 20–25 cm by using a piece of adhesive tape wrapped around the tail 1 cm from the tip. Behaviour was recorded for 6 min. The duration of immobility was calculated by an observer blinded to the treatment groups. The mice were considered to be immobile only when they remained completely motionless; mice that climbed along their tails were not included.

#### Forced swimming test

The FST was performed as previously described [86], in a clear glass cylinder filled with water (temperature, 23–25 °C); cylinder’s dimensions were: height, 30 cm; diameter, 20 cm; water level, 15 cm. Mice were gently placed in the tanks. Behaviour was recorded for 6 min. The movement of the animals was video recorded and analysed later. Following the swimming session, the mice were removed from the water by their tails, gently dried with towels, and kept warm under a lamp in their home cages. They were considered to be immobile whenever they stopped swimming and remained floating passively, still keeping their heads above the surface of the water.

#### Open field test

The open field test was performed as previously described [86]. The apparatus consisted of a rectangular chamber (50 × 50 × 50 cm) made of white, high-density, non-porous plastic. Mice were gently placed in the centre of the chamber and their motility was recorded for 10 min. The total running distance, and the time spent in the centre versus the periphery of the open field chamber were recorded by a camera connected to a computer using an automated video tracking program (EthoVision XT 9.0; Noldus, Wageningen, The Netherlands). The chamber was thoroughly cleaned with 95% ethanol, and dried prior to use and before subsequent tests, to remove any scent clues left by the previous subject.

### AAVs microinjections

Viral injections were performed at the end of week 3 of CUMS treatment as indicated in Fig. 1 by using a stereotaxic apparatus (RWD, Shenzhen, China) to guide the placement of a Hamilton syringe fixed with bevelled glass pipettes (Sutter Instrument, 1.0-mm outer diameter) into the PFC [88]. The injection site was located at half of the distance along a line defined between each eye and the lambda intersection of the skull. The needle was held perpendicular to the skull surface during insertion to a depth of approximately 0.2 mm. A total of 0.7 μl of AAV5-gfaABC1D-mCherry (1 × 10^12^ gc/mL; Taitool Bioscience, Shanghai, China), was slowly injected into right sides of the PFC. Glass pipettes were left in place for at least 5 min. After injection, animals were allowed to completely recover under a warming blanket and then returned to the home cage.

### Immunohistochemistry

Mice were perfused in the morning with cold paraformaldehyde (PFA, 4% w/v in phosphate buffer saline (PBS)) under deep isoflurane (2%, 5 min) and pentobarbitone (1%, 50 mg/kg) anaesthesia. Brains were collected, postfixed and cryoprotected in 30% (w/v) sucrose solution. Brains were cut using a cryostat in 45 um thick sections; slices were immediately transferred into storing solution (30% w/v sucrose and 30% ethylene glycol in PBS) and kept at 80 °C until use. Free-floating sections were incubated 1 h in saturation solution (6% fetal calf serum in PBS). The sections were then incubated overnight in the same solution complemented with the primary antibody (rabbit anti-Ezrin 1:100 CellSignalling, Danvers, Massachusetts, USA). After washing in PBS three times, slices were incubated 1 h at 37 °C in saturation solution containing the relevant secondary antibody (goat anti-rabbit Alexa 488; Invitrogen, Carlsbad, California, USA). After washing in PBS three times, labelling nucleus with DAPI, the coverslips were mounted on slides using anti-fade solution (Solarbio, Beijing, China). Confocal microscopy (Olympus, Tokyo, Japan) or normal fluorescence microscope (Leica, Wetzlar, Germany) were used to obtain images.

### Sholl analysis

Sholl analysis is a commonly used method to quantify astrocyte process complexity [43, 54]. All processing steps were performed using image analysis software ImageJ [https://imagej.net/imagej-wiki-static/Sholl_Analysis]. In brief, Z-stacks corresponding to the emission spectrum (565–610 nm) of mCherry-labelling (resolution was 512 × 512 pixels (0.2 μm/px) on XY axis with a step on Z-axis 1 μm/frame were re-sampled to the same lateral resolution of 0.25 μm/px.

### 3D reconstructions

The confocal imaging stacks were collected with a Z-step size of 0.25 μm under a confocal microscope (Olympus, Tokyo, Japan). Three-dimensional reconstructions were processed offline using Imaris 7.4.2 (Bitplane, South Windsor, CT) as reported previously [89]. In brief, the astrocyte soma and processes were measured and reconstructed according to their own parameter. Processes diameter was measured as one-tenth of astrocyte soma. In addition, Ezrin was measured as 1 mm in every group. The surface–surface colocalisation was calculated by a specific plugin of Imaris [40].

### Statistics

All statistical analyses were performed by GraphPad Prism 8. All data were expressed as means ± SEM of n observations, where n means the number of animals in behavioural tests, or astrocyte cells from at least three animals. All analysis was single-blind. Data with more than two groups were tested for significance using one-way ANOVA test followed by the Holm–Sidak test. Multiple comparisons between the data were performed in case of their non-normal distribution, using the Kruskal–Wallis ANOVA on ranks, followed by Tukey’s test. A two-way ANOVA followed by Dunn’s test was performed to compare data obtained in Figs. 2I, 3B, 6B. Significance was defined as *P* < 0.05.

## Supplementary information

Reproducibility checklist

## Data Availability

The datasets used and analysed are available from the corresponding authors upon reasonable request.
